# Optimization of Solid-Supported Glaser-Hay Reactions in the Microwave

**DOI:** 10.3390/molecules20045276

**Published:** 2015-03-24

**Authors:** Jessica S. Lampkowski, Johnathan C. Maza, Sanjana Verma, Douglas D. Young

**Affiliations:** Department of Chemistry, College of William & Mary, P.O. Box 8795, Williamsburg, VA 23187, USA; E-Mails: jslampkowski@email.wm.edu (J.S.L.); jcmaza@email.wm.edu (J.C.M.); sverma01@email.wm.edu (S.V.)

**Keywords:** microwave irradiation, solid-support, Glaser-Hay

## Abstract

The translation of organometallic reactions into a microwave reactor has numerous advantages. Herein, we describe the application of a previously developed solid-supported Glaser-Hay reaction to microwave conditions. Overall, an array of diynes has been prepared demonstrating the ability to conduct chemoselective reactions in the microwave within 20 min compared to the 16 h thermal conditions. Moreover, non-microwave transparent alkynes have been found to react more quickly, preventing catalyst quenching, and resulting in higher yields.

## 1. Introduction

**Figure 1:**
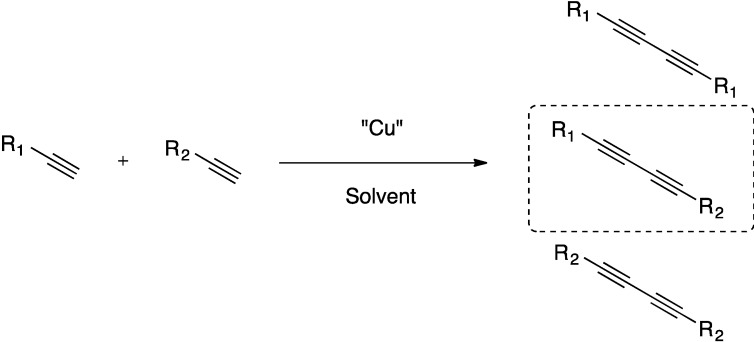
Chemoselectivity of the Glaser-Hay reaction.

The Glaser-Hay reaction involves the coupling of terminal alkynes to afford polyynes in good to excellent yields [1,2,3,4]. These polyynes are found in various applications ranging from biologically active natural products to optical materials [5,6,7,8]. A substantial hurdle to the widespread application of the Glaser-Hay reaction in synthetic organic chemistry is its lack of chemoselectivity, as the coupling of two unique terminal alkynes typically results in a mixture of homodimer and heterodimer products (Figure 1). While some chemoselective alkyne couplings have been elucidated, the simplicity of the Glaser-Hay reaction makes it a desirable choice in the synthesis of polyynes [9,10,11,12,13,14,15]. To this end, we have recently reported the chemoselective Glaser-Hay reaction via the immobilization of one terminal alkyne [16,17]. Immobilization of the alkyne precludes its homodimerization, and dimerization of the soluble alkyne can be rapidly washed away. This affords a facile synthesis of the heterodimeric product in good yields and excellent purities. 

While the previous chemoselective reaction involving the solid support is useful, the 16 h reaction time is synthetically limiting when preparing large libraries of molecules. To overcome this issue, we set forth to investigate the potential rate enhancement that could be obtained by the utilization of microwave chemistry. Microwave irradiation induces molecular motion via the alignment of ions or dipoles with the oscillating electromagnetic field, and has been demonstrated to increase reaction yields and to decrease reaction times [18,19]. Because the microwaves interact directly with the reagents and solvent, instead of by convection, they afford efficient heating of the reactions, cleaner reaction conditions, and in some cases, facilitate reactions that are not achievable by conventional thermal heating [20,21]. By translating the previously described Glaser-Hay reaction to the microwave, we aim to increase its synthetic utility even further. Previous research has successfully translated a Glaser-Hay macrocyclization to the microwave, resulting in the standard reaction time of 48 h to be decreased to 1–6 h [22]. Similarly, we aim to decrease the reaction times of the reported solid-supported Glaser-Hay coupling from 16 h to 10–20 min. 

## 2. Results and Discussion

### 2.1. Optimization of Microwave Conditions 

Translation of the previously developed solid-supported Glaser-Hay reaction to the microwave was initiated utilizing a propargyl alcohol immobilized resin and phenylacetylene as a soluble alkyne due to the rapid monitoring of the reaction via TLC analysis and UV detection. While other solvents including DCM, acetone, and toluene were briefly investigated, THF was found to be optimal under microwave conditions. This correlated to the previously optimized thermal conditions and was ideal due to the microwave transparency of THF. Microwave conditions were assessed under varying irradiation times in both power mode (100, 200 and 300 W) and in temperature mode with set temperatures. Interestingly, when attempted in power mode at any setting for 5–20 min, little diyne product was detected. When examining the temperature profiles, temperatures ranging from 140–180 °C were observed. At these temperatures it is possible that resin degradation may be occurring, resulting in diminished yields. Consequently, we shifted our focus to temperature mode, which involves pre-setting a maximum temperature the reaction can reach, and varying the reaction times. Temperatures between 40 °C and 100 °C were examined with irradiation times of 5–20 min (Table 1).

**Table 1:** Microwave Optimization of the Glaser-Hay Reaction. 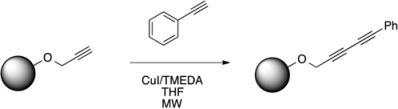

Temperature (°C)	Time (min)	Yield (%)
40	20	0
60	5	26
60	10	74
80	5	12
80	10	77
80	20	77
100	5	0
100	10	63

Based on these preliminary results, it appears that a minimum of 10 min of reaction time is required in order to yield significant product independent of temperature conditions. Prolonged reaction times did not increase product yield. Optimal conditions were observed when irradiating for 10 min at 80 °C, affording a 77% yield after resin cleavage. Increased temperatures resulted in decreased yields perhaps due to resin or catalyst degradation under microwave conditions. However, these conditions represent a significant time enhancement relative to the previously developed thermal conditions that required 16 h of reaction time.

### 2.2. Comparison to Thermal Conditions 

With optimized conditions in hand, the direct comparison of thermal conditions to the microwave was assessed. Using a series of commercially available alkynes, an array of diynes was prepared and their yields were compared to that obtained by thermal reaction at 60 °C for 16 h. Reactions were performed with alkynes harboring a range of chemical functionality including aromatic rings (**1**, **3**, and **4**), aliphatic chains (**2**), ethers (**3**), basic residues (**4**, **5**), and alcohols (**6**) with the propargyl alcohol derivatized resin to yield a series of diynes (**7**–**12**). Interestingly, the previous conditions were not found to be optimal for all soluble alkynes, perhaps due to their differential absorbance of microwave irradiation. Thus, increasing reaction times to 20 min seemed to afford maximal results and enhanced yields (Table 2). 

**Table 2:** Propargyl Alcohol Derivatized Resin Glaser-Hay Reactions. 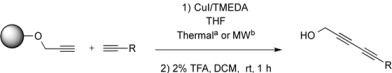

Alkyne	Product	% Yield Thermal ^a^	%Yield Microwave ^b^
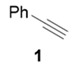	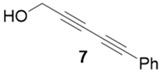	95%	77%
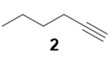	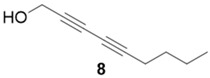	84%	67%
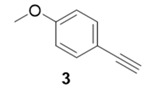	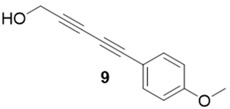	99%	75%
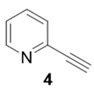	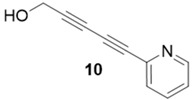	55%	90%
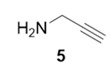	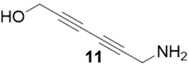	40%	92%
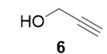	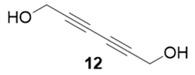	99%	90%

^a^ 60 °C, 16 h; ^b^ 80 °C, 20 min.

The assembly of the diyne library demonstrated several important factors must be considered when using the microwave in this technology. Overall, microwave yields were good to excellent (67%–92%), but were highly dependent on the soluble alkyne. In the case of incomplete reaction, starting materials were removed via filtration through a silica plug. Alkynes with basic, coordinating moieties (**4**–**5**) were obtained in higher yields in the microwave relative to the thermal conditions. We hypothesize this is due to the rate enhancement in the microwave affording catalysis prior to catalyst poisoning by soluble alkyne coordination to the copper catalyst. Additionally, somewhat nonpolar alkynes (**1**–**3**) appear to be less reactive under microwave irradiation, resulting in lower yields than thermal conditions. Attempts to extend reaction times in these cases did not result in any increase of diyne product. 

Due to the differences in soluble alkynes, we also investigated the influence of the immobilized alkyne via derivitization of the trityl chloride resin with 4-ethynyl benzyl alcohol. This resin was then reacted with the same set of soluble alkynes employed with the propargyl alcohol resin. Using the previously described microwave conditions resulted in substantial amounts of unreacted alkyne on the resin, requiring further optimization of conditions. Ultimately, this resin was found to be highly adsorbing of microwave irradiation, heating much more rapidly than the propargyl alcohol resin. This resulted in very low microwave input into the reactions. To rectify this, the reaction was subjected to 2 min pulses at 120 °C for a total of 20 min (Table 3). Each pulse heats the reaction to 120 °C for a 2-min interval, followed by cooling to room temperature prior to resubmission to microwave irradiation. 

**Table 3:** Ethynyl Benzyl Alcohol Derivatized Resin Glaser-Hay Reactions. 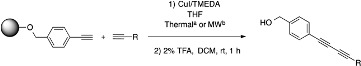

Alkyne	Product	% Yield Thermal ^a^	%Yield Microwave ^b^
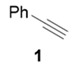	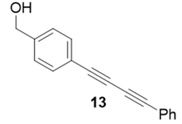	96%	78%
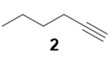	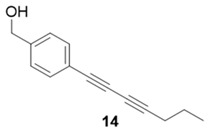	98%	76%
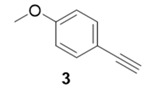	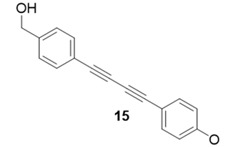	73%	76%
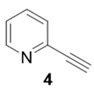	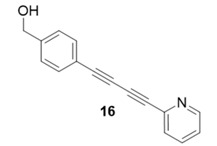	53%	86%
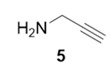	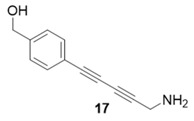	96%	86%
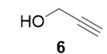	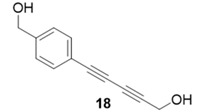	40%	86%

^a^ 60 °C, 16 h; ^b^ 120 °C, 2 min × 10 pulses.

The series of diynes (**13**–**18**) were obtained in good to excellent yield, ranging from 76%–86%. Similar to results obtained with the propargyl alcohol resin, soluble terminal alkynes with a degree of polarity afforded higher yields under microwave conditions than observed under thermal conditions. Also, the ethynyl benzyl alcohol resin demonstrated the need to optimize reaction conditions for alkynes of differing reactivity and microwave transparency. Interestingly, the thermal yields of the reaction with the catalyst coordinating alkyne **5** were higher, unlike reactions observed on the propargyl alcohol resin. This observation may be reflective of the decreased reactivity of the ethynyl benzyl alcohol resin no longer aiding the previously observed microwave enhancement in the presence of this type of soluble alkyne.

## 3. Experimental Section

### 3.1. General

Solvents and reagents were obtained from either Sigma-Aldrich (St. Louis, MO, USA) or Fisher Scientific (Waltham, MA, USA) and used without further purification, unless noted. Tritylchloride resin, 100–200 mesh, 1% DVB crosslinking, was purchased from Advanced Chemtech. (Louisville, KY, USA) Microwave reactions were performed in a CEM Discover System. Reactions were conducted under ambient atmosphere with non-distilled solvents. NMR data was acquired on a Varian Gemini 400 MHz. Compound purities were assessed by NMR and found to be 90% or greater for all compounds.

### 3.2. Alkyne Immobilization Protocol

Trityl chloride resin (200 mg, 0.36 mmol, 1 equiv.) and dichloromethane (5 mL) were added to a flame-dried vial. The resin was swelled at room temperature with gentle stirring for 15 min. The alkyn-ol (~1.2 equiv.) was added to reaction, followed by triethylamine (10.0 µL, 0.072 mmol, 0.2 equiv.). The mixture was stirred at room temperature for 16 h. The resin was transferred to a syringe filter and washed with DCM and MeOH (5 alternating rinses with 5 mL each). The resin was swelled in CH_2_Cl_2_ and dried under vacuum for 45 min before further use.

### 3.3. General Glaser-Hay Coupling Protocol

Soluble alkyne (0.70 mmol, 10 equiv.) was added to a flame dried microwave vial containing the propargyl alcohol derivitized trityl resin (100 mg, 0.07 mmol, 1 equiv.), and tetrahydrofuran (2.0 mL). Copper iodide (10 mg, 0.53 mmol.) and tetramethylethylenediamine (50 µL, 0.33 mmol.) were added to a separate flame-dried vial then dissolved in tetrahydrofuran (2.0 mL). The catalyst mixture was then added to the resin in one portion and placed in the microwave reactor to run for the allotted time in temperature mode at a specific temperature. The completed reaction was transferred to a syringe filter and washed with DCM and MeOH (5 alternating rinses with 5 mL each). The product was then cleaved from the resin by treatment with 2% TFA (DCM, 1 mL, 1 h) and filtered into a vial. Product was analyzed via ^1^H-NMR and GCMS.

### 3.4. Analytical Data

**Compound 7**: same as below: The solvent was removed *in vacuo* to give compound 7 as a solid (6 mg, 0.039 mmol, 77%). ^1^H-NMR (400 MHz; CDCl_3_): ∂ 7.49 (t, *J* = 5.9, 2H), 7.34–7.26 (m, 3H), 4.45 (s, 2H), 1.94 (s, 1H); GCMS (R_t_ = 9.20 min) calculated for C_11_H_8_O 156.1, found 156.2. 

**Compound 8**: The solvent was removed *in vacuo* to give compound 8 as a solid (5 mg, 0.034 mmol, 67%) ^1^H-NMR (400 MHz; CDCl_3_): ∂ 4.32 (s, 2H), 2.29 (t, *J* = 6.8 Hz, 3H), 1.52 (m, 2H), 1.42 (sextet, *J* = 7.2 Hz, 2H), 0.91 (t, *J* = 7.23 Hz, 3H); GCMS (R_t_ = 7.29 min) calculated for C_9_H_12_O 136.1, found 136.0.

**Compound 9**: The solvent was removed *in vacuo* to give compound 9 as a solid (7 mg, 0.038 mmol, 75%) ^1^H-NMR (400 MHz; CDCl_3_): ∂ 7.44 (d, *J* = 7.6 Hz, 2H), 6.85 (d, *J* = 7.6, 2H), 4.14 (s, 2H), 3.82 (s, 3H); GCMS (R_t_ = 9.93 min) calculated for C_12_H_10_O_2_ 186.1, found 186.2.

**Compound 10**: The solvent was removed *in vacuo* to give compound 10 as a solid (7 mg, 0.045 mmol, 90%). ^1^H-NMR (400 MHz; CDCl_3_): ∂ 8.01 (d, *J* = 7.5 Hz, 1H), 7.65 (t, *J* = 7.4 Hz, 1H), 7.32 (t, *J* = 7.5 Hz, 1H), 6.91 (d, *J* = 7.4 Hz, 1H), 4.38 (s, 2H). GCMS (R_t_ = 8.77 min) calculated for C_10_H_7_NO 157.1, found 157.2.

**Compound 11**: The solvent was removed *in vacuo* to give compound 11 as a solid (5 mg, 0.046 mmol, 92%). Using 7 as a starting material (3.5 mg, 0.032 mmol, 91%). ^1^H-NMR (400 MHz; CD_3_OD): ∂ 5.21 (s, 2H), 4.35 (s, 2H), 3.39 (s, 2H); GCMS (R_t_ = 6.01 min) calculated for C_6_H_7_NO 109.1, found 109.1.

**Compound 12**: The solvent was removed *in vacuo* to give compound 12 as a solid (5 mg, 0.045 mmol, 90%). ^1^H-NMR (400 MHz; CDCl_3_): ∂ 4.36 (s, 4H); GCMS (R_t_ = 7.88 min) calculated for C_6_H_6_O_2_ 110.0, found 110.1. 

**Compound 13**: The solvent was removed *in vacuo* to give compound 13 as a solid. (9 mg, 0.039 mmol, 78%). ^1^H-NMR (400 MHz; CDCl_3_): δ 7.53 (d, *J* = 7.5 Hz, 4H), 7.45 (t, *J* = 7.5 Hz, 4H), 7.30 (m, *J* = 7.5, 5H), 4.74 (s, 2H); GCMS (R_t_ = 10.75 min) calculated for C_17_H_12_O_2_ 232.1, found 232.1.

**Compound 14**: The solvent was removed *in vacuo* to give compound 14 as a solid. (4 mg, 0.019 mmol, 38%). ^1^H-NMR (400 MHz; CDCl_3_): δ 7.52 (d, *J* = 7.5 Hz, 2H), 7.31 (d, *J* = 7.4 Hz, 2H), 4.73 (s, 2H), 2.32 (t, *J* = 7.1 Hz, 2H), 1.56 (quintet, *J* = 7.1 Hz, 2H), 1.42 (m, 2H), 0.95 (t, *J* = 7.1 Hz, 3H); GCMS (R_t_ = 9.85 min) calculated for C_15_H_16_O 212.1, found 212.2. 

**Compound 15**: The solvent was removed *in vacuo* to give compound 15 as a solid. (10 mg, 0.038 mmol, 76%). ^1^H-NMR (400 MHz; CDCl_3_): δ 7.53 (d, *J* = 7.5 Hz, 2H), 7.30 (d, *J* = 7.5 Hz, 2H), 7.40 (d, *J* = 7.5 Hz, 2H), 6.95 (d, *J* = 7.5 Hz, 2H), 4.65 (s, 2H), 3.96 (s, 3H); GCMS (R_t_ = 13.10 min) calculated for C_18_H_14_O_2_ 262.1, found 262.1. 

**Compound 16**: The solvent was removed *in vacuo* to give compound 16 as a solid. (10 mg, 0.043 mmol, 86%). ^1^H-NMR (400 MHz; CDCl_3_): δ 7.61 (t, *J* = 8.6 Hz, 1H), 7.55 (t, *J* = 8.6 Hz, 1H), 7.53 (d, *J* = 7.3 Hz, 2H), 7.40 (d, *J* = 7.4 Hz, 1H), 7.31 (d, *J* = 7.3 Hz, 2H), 7.25 (s, 1H), 4.65 (s, 2H); GCMS (R_t_ = 10.75 min) calculated for C_6_H_11_NO 233.1, found 233.3.

**Compound 17**: The solvent was removed *in vacuo* to give compound 17 as a solid. (8 mg, 0.043 mmol, 86%). ^1^H-NMR (400 MHz; CDCl_3_): δ 7.40 (d, *J* = 7.5 Hz, 2H), 7.33 (d, *J* = 7.5 Hz, 2H), 4.61 (s, 2H), 3.28 (s, 2H). GCMS (R_t_ = 9.92 min) calculated for C_12_H_11_NO 185.1, found 185.2. 

**Compound 18**: The solvent was removed *in vacuo* to give compound 18 as a solid (8 mg, 0.043 mmol, 86%). ^1^H-NMR (400 MHz; CDCl_3_): ∂ 7.49 (d, *J* = 8.0 Hz, 2H), 7.44 (d, *J* = 8.0 Hz, 2H), 4.72 (s, 2H), 4.35 (s, 2H); GCMS (R_t_ = 9.94 min) calculated for C_12_H_10_O_2_ 186.1, found 186.1.

## 4. Conclusions

In conclusion, the utilization of microwave irradiation has been found to increase the reaction rate of solid-supported Glaser-Hay reactions, improving their utility in the preparation of large combinatorial libraries via a more efficient synthesis. Moreover, the polarity of the diyne was found to be an important factor in reaction rate, as more polar alkynes were obtained in higher yields relative to their microwave transparent counterparts. Moreover, alkynes with basic, coordinating moieties were often found to perform better in the microwave than under thermal conditions as reduced catalyst poisoning was observed due to enhanced reaction rates. Overall, the microwave mediated, solid-supported Glaser-Hay reaction represents an efficient synthetic methodology to chemoselectively obtain a range of heterodimeric polyynes that can be employed in a variety of applications.

## References

[1] Vilhelmsen M., Jensen J., Tortzen C., Nielsen M. (2013). The Glaser-Hay Reaction: Optimization and Scope Based on C-13 NMR Kinetics Experiments. Eur. J. Org. Chem..

[2] Glaser C. (1896). Beitrage zur Kenntiniss des Acetyenylbenzols. Ber. Dtsch. Chem. Ges..

[3] Hay A.S. (1962). Polymerization by oxidative coupling.II. Oxidation of 2, 6-disubstituted phenols. J. Poly. Sci..

[4] Hay A.S. (1962). Oxidative Coupling of Acetylenes. J. Org. Chem..

[5] Wong W. (2005). Recent advances in luminescent transition metal polyyne polymers. J. Inorg. Organomet. Polym. Mater..

[6] Shi Shun A.L.K., Tykwinski R.R. (2006). Synthesis of naturally occurring polyynes. Angew. Chem. Int. Ed..

[7] Slepkov A.D., Hegmann F.A., Eisler S., Elliott E., Tykwinski R.R. (2004). The surprising nonlinear optical properties of conjugated polyyne oligomers. J. Chem. Phys..

[8] Pan Y., Lowary T., Tykwinski R.R. (2009). Naturally occurring and synthetic polyyne glycosides. Can. J. Chem. Rev..

[9] Klebansky A., Dolgopolsky I., Dobler Z. (1957). The Role of Complex Compounds and Cations of Complex-Forming Components in the Polymerization of Acetylene. Dokl. Akad. Nauk SSSR.

[10] Yin W., He C., Chen M., Zhang H., Lei A. (2009). Nickel-Catalyzed Oxidative Coupling Reactions of Two Different Terminal Alkynes Using O-2 as the Oxidant at Room Temperature: Facile Syntheses of Unsymmetric 1,3-Diynes. Org. Lett..

[11] Montierth J., DeMario D., Kurth M., Schore N. (1998). The polymer-supported Cadiot-Chodkiewicz coupling of acetylenes to produce unsymmetrical diynes. Tetrahedron.

[12] Chodkiewicz W., Cadiot P., Willemart A. (1957). Diethynyl-Arenes. Comptes Rendus Hebd. Seances Acad. Sci..

[13] Zheng Q., Hua R., Wan Y. (2010). An Alternative CuCl—Piperidine-Catalyzed Oxidative Homocoupling of Terminal Alkynes Affording 1,3-Diynes in Air. Appl. Organomet. Chem..

[14] Wang L., Yan J., Li P., Wang M., Su C. (2005). The Effect of Amines on Oxidative Homo-Coupling of Terminal Alkynes Promoted by Copper Salts. J. Chem. Res..

[15] Li L., Wang J., Zhang G., Liu Q. (2009). A Mild Copper-Mediated Glaser-Type Coupling Reaction Under the Novel CuI/NBS/DIPEA Promoting System. Tetrahedron Lett..

[16] Lampkowski J.S., Durham C.E., Padilla M.S., Young D.D. (2014). Preparation of Asymmetrical Polyynes by a Solid-Supported Glaser-Hay Reaction. Org. Biomol. Chem..

[17] Tripp V.T., Lampkowski J.S., Tyler R., Young D.D. (2014). Development of Solid-Supported Glaser-Hay Couplings. ACS Comb. Sci..

[18] Herrero M.A., Kremsner J.M., Kappe C.O. (2008). Nonthermal Microwave Effects Revisited: On the Importance of Internal Temperature Monitoring and Agitation in Microwave Chemistry. J. Org. Chem..

[19] Hoz A, Loupy A (2012). Microwaves in Organic Synthesis.

[20] Senaiar R., Young D.D., Deiters A. (2006). Pyridines via Solid-Supported [2 + 2 + 2] Cyclotrimerization. Chem. Commun..

[21] Young D.D., Deiters A. (2007). A General Approach to Chemo- and Regioselective Cyclotrimerization Reactions. Angew. Chem. Int. Ed..

[22] Bédard A.C., Collins S.K. (2012). Microwave Accelerated Glaser-Hay Macrocyclizations at High Concentrations. Chem. Commun..

